# Improved Drain Current Saturation and Voltage Gain in Graphene–on–Silicon Field Effect Transistors

**DOI:** 10.1038/srep25392

**Published:** 2016-05-04

**Authors:** Seung Min Song, Jae Hoon Bong, Wan Sik Hwang, Byung Jin Cho

**Affiliations:** 1 Department of Electrical Engineering, KAIST, Daejeon, 305-338 Korea; 2 Department of Materials Engineering, Korea Aerospace University, Goyang, 412-791, Korea

## Abstract

Graphene devices for radio frequency (RF) applications are of great interest due to their excellent carrier mobility and saturation velocity. However, the insufficient current saturation in graphene field effect transistors (FETs) is a barrier preventing enhancements of the maximum oscillation frequency and voltage gain, both of which should be improved for RF transistors. Achieving a high output resistance is therefore a crucial step for graphene to be utilized in RF applications. In the present study, we report high output resistances and voltage gains in graphene-on-silicon (GoS) FETs. This is achieved by utilizing bare silicon as a supporting substrate without an insulating layer under the graphene. The GoSFETs exhibit a maximum output resistance of 2.5 MΩ∙μm, maximum intrinsic voltage gain of 28 dB, and maximum voltage gain of 9 dB. This method opens a new route to overcome the limitations of conventional graphene-on-insulator (GoI) FETs and subsequently brings graphene electronics closer to practical usage.

The enormous interest in graphene for electronic device applications originates from its outstanding charge transport properties and atomic-scale thickness^1^,^2^,^3^. However, graphene devices suffer from poor on/off current ratios, which are caused by a zero band gap property. This limits their applications in logic transistors. Nevertheless, owing to graphene’s exceptionally high carrier mobility and saturation velocity, it is a strong candidate for RF device applications, since RF transistors do not have to be switched off in operating conditions^1^,^4^,^5^.

Intensive research activities examining graphene RF FETs thus far have mainly focused on achieving higher cut-off frequencies (*f*_*T*_) with the help of gate length scaling^6^. Recently, *f*_*T*_ values as high as 400 GHz were reported from state-of-the-art graphene RF FETs^7^,^8^,^9^,^10^,^11^,^12^,^13^,^14^,^15^,^16^. However, from a circuit designer’s point of view, rather than *f*_*T*_, the maximum oscillation frequency (*f*_*max*_) and intrinsic voltage gain (*A*_V0_) are more important factors for RF devices^1^, and very recently *f*_*max*_ values as high as 105 GHz were reported^17^. The poor f_max_ and A_*V*0_ values of conventional GoIFETs mainly originate from the absence of current saturation in the output characteristics due to the absence of a band gap and the Klein tunneling effect^18^.

It is therefore highly desirable to obtain robust current saturation in the output characteristics from graphene FETs, which would result in a higher *f*_*max*_ and *A*_*V*0_^19^,^20^,^21^. FET current saturation can be observed, either due to the decrease of carrier density in the channel near the drain side or to the velocity saturation of the carriers caused by phonon scattering^22^. For non-zero band gap semiconductors such as Si, a depletion region is formed at the drain side under *V*_*GS*_ − *V*_*TH*_ = *V*_*DS*_, and the width of the depletion region extends as *V*_*DS*_ increases, where *V*_*GS*_ is the gate-to-source voltage, *V*_*TH*_ is the threshold voltage, and *V*_*DS*_ is the drain-to-source voltage. On the other hand, zero band gap materials such as graphene do not form depletion regions in the channel. Rather than forming a depletion region, a different polarity charge is generated at the drain side, and this charge extends as *V*_*DS*_ increases. This generation of other charged carriers in the channel leads to the presence of a kink-like feature in the output curve of the GoIFETs, resulting in lower output resistance (*r*_*o*_) and thereby degraded RF performance. Thus, by suppressing the generation of the other charged carrier in the channel near the drain, the graphene device is able to obtain a higher *r*_*o*_.

One of the methods that has been attempted to suppress the generation of the other charged carriers is the strengthening of the gate electrostatic control. In other words, the influence of the drain voltage is weakened with respect to the graphene channel near the drain side. Thus, previous attempts have focused on increasing the gate coupling by increasing the gate capacitance^19^,^20^,^21^ and/or minimizing the residual carrier concentration (*n*_0_)^22^,^23^.

Recently, hexagonal boron nitride (*h*-BN) has been adopted as a bottom gate dielectric to minimize *n*_0_^22^,^23^ since *h*-BN exhibits lower charged impurities and provide higher optical phonon energy than a conventional SiO_2_ substrate^19^,^23^. The minimum *n*_0_, which was proportional to the charged impurities, disorder, and thermal excitation, was found to significantly affect the drain conductance (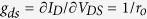)^24^,^25^. Despite the importance of obtaining current saturation in the output characteristics, robust current saturation has not yet been demonstrated in graphene FETs that offer highly compatible integration with current transistor processes.

In this study, we present simple but improved output characteristics by utilizing silicon instead of conventional SiO_2_ or *h*-BN as a substrate. The implementation of Si as a substrate material for graphene has additional advantages over *h*-BN and other insulators in that the integration in wafer-sized scales is comparable to conventional FET technologies. Several other groups have also reported that graphene transfers onto Si surfaces, but their studies focused on the formation of a Schottky junction to modulate the current, which entails charge transport vertically from the graphene to silicon^26^,^27^,^28^. Moreover, their devices still suffered from poor output resistance. In contrast to previous approaches^28^, in this work the charged carriers only move through the graphene layer. In other words, the current flows in a horizontal direction parallel to the silicon surface, and the carriers are confined within the graphene. The proposed device shows improved output resistance.

The typical architecture of a GoSFET is shown in Fig. 1a. The only difference between the GoSFET and conventional GoIFETs is the absence of an oxide layer under the graphene. Due to the lack of an insulating layer under the graphene in GoSFETs, the potential in the graphene is strongly affected by the charge carrier density and carrier type of the Si substrate. Figure 1b shows typical GoSFET transfer characteristics at both *V*_*DS*_ = 1 V and *V*_*DS*_ = −1 V, where lightly doped n-type silicon was used as a substrate. Excellent transfer characteristics were observed under *V*_*DS*_ = −1 V, where the charge in the Si was depleted at the drain side due to the reverse bias condition, as shown in the inset of Fig. 1b. Unlike the reverse bias condition at *V*_*DS*_ = −1 V, the performance of the GoSFET degraded under *V*_*DS*_ = 1 V due to the non-negligible contribution of the body current, *I*_*B*_, which was caused by the forward bias condition. A further investigation of *I*_*B*_, depending on the type of Si substrate, was conducted, and the results are shown in Fig. 1c,d. In Fig. 1c, *I*_*B*_ is 10^5^ times lower than the drain current (*I*_*D*_), indicating that the silicon substrate successfully acted as an insulator under the reverse bias condition. In contrast to the reverse bias condition at *V*_*DS*_ < 0 (Fig. 1c), the electrons in the body flowed to the graphene, and *I*_*B*_ became comparable to *I*_*D*_ under the forward bias condition at *V*_*DS*_ > 0 (Fig. 1d). *I*_*B*_ should be suppressed in the output characteristics. The doping type of the Si substrate thus should be considered depending on the operating voltage conditions to form a depletion region in the channel near the drain side, as shown in the inset of Fig. 1d.

Figure 2a shows typical GoSFET output characteristics, revealing improved current saturation in a wide drain voltage range. Unlike the improved current saturation in the GoSFETs, current saturation in a narrow drain voltage range was observed in the output characteristics of the conventional GoIFETs in Fig. 2b, which is typical in graphene FETs. The contour plots of *g*_*ds*_ in Fig. 2c,d clearly show that the current saturation of the GoSFETs is much more robust than that of the conventional GoIFETs. The improved drain current saturation in the GoSFETs results in a lower *g*_*ds*_ and higher output resistance, as shown in Fig. 2e. The maximum *r*_*o*_ at a given V_GS_ is plotted in Fig. 2f, showing that a *r*_*o*_ value as high as 2.5 MΩ∙μm was obtained from the GoSFETs, in contrast to the *r*_*o*_ value of 0.12 MΩ∙μm obtained from the GoIFETs. This improved GoSFET drain current saturation is mainly attributed to the suppression of electron carrier generation as the drain voltage increases, which is described in Fig. 3b,c. The potential energy of the drain is coupled with the energy level of the Si substrate. Thus, the positive fixed charge is generated in the depletion region near the drain side. This additional potential from the Si substrate allows a higher graphene energy level and thus the graphene is able to maintain its p-type behavior even at higher drain voltages. On the other hand, in the GoIFETs, other polarity carriers are generated near the drain side at higher drain voltages, which causes the kink effect in the GoIFETs, as shown in Fig. 3a–c. It is worth noting that the current takeoff at high drain voltage in the GoSFET is steeper than that in the GoIFET. The surface potential at the onset of the strong inversion in the Si at high drain voltage leads to a hole inversion layer, generating a mobile carrier charge and eventually contributing to the drain current, as shown in Fig. 3d.

The cumulative results of the maximum *r*_*o*_ in Fig. 4a clearly show that, in terms of the RF characteristics, the GoSFETs outperformed the conventional GoIFETs due to the higher *r*_*o*_. We further investigated the correlation between the maximum *r*_*o*_ and several device parameters, including the residual carrier concentration, *n*_0_, contact resistance, *R*_*C*_, and FET carrier mobility, *μ*, as shown in Fig. 4b–d. Their values were extracted from the fitting using the model (Equation (1) in the supplementary information) for both the GoSFETs and GoIFETs^29^. It was found that both *n*_0_ and *R*_*C*_ were inversely proportional to the maximum *r*_*o*_ in logarithmic scale, especially for the GoSFETs, as shown in Fig. 4b,c. The robust current saturation required strong gate coupling, which could be obtained at low parasitic capacitance, i.e., a low *n*_0_^17^,^24^. Generally, low GoSFET values are attributed to the non-polar property of the silicon surface, which suppresses residual impurities between the graphene and the silicon surface. Unlike on the silicon surface, charged residues can easily attach to the SiO_2_ surface (polar behavior), which results in the doping of the graphene and degradation of the graphene devices^30^. The residual charges on the SiO_2_ were reported to be around 2 ~ 5 × 10^11 ^cm^−2^, while those on the silicon surface were around 0.3 ~ 2.8 × 10^10^ cm^−2 ^31,32, which were also verified by the Raman analysis as shown in the supplementary (Fig. S2). The experimental results indicated that there are more residual charges on graphene/SiO_2_ than graphene/Si interfaces.

The suppression capabilities that prevent the generation of the other polarity carrier in the channel near the drain are directly affected by the electron potential energy in the Si, which is influenced by the metal/graphene voltage drop. Therefore, it is expected that the maximum *r*_*o*_ is inversely proportional to *R*_*C*_ for the GoSFETs, while *r*_*o*_ exists irrespective of *R*_*C*_ for the GoIFET. The lower contact resistance in the GoSFETs compared to that of the GoIFETs is attributed to the presence of additional charge injection into the graphene from the Si bottom substrate, in addition to the metal pad. Both Pd and Si atoms are able to inter-diffuse through the defects in the graphene, such as carbon atom vacancies, thereby form a conductive Pd silicide (Fig. S1) in the Si substrate^33^, which is the source of the additional charge injection into the graphene. The lower contact resistance of the graphene devices is inter-correlated with the higher FET mobility in the GoSFETs. Finally, a weak correlation between the maximum *r*_*o*_ and *μ* can be observed in Fig. 3d. Nevertheless, it is evident that the FET mobility of GoSFETs is higher than that of GoIFETs due to the higher phonon energy of Si (63 meV) compared to that of SiO_2_ (50 meV)^34^,^35^.

Figure 5a shows that the maximum *r*_*o*_ was proportional to the maximum A_*V*0_, and that a maximum A_*V*0_ as high as 28 dB was achieved from the GoSFETs, which is comparable to that of Si-based RF FETs. In order to verify the performance of the voltage amplifier, a common-source voltage amplifier was implemented with a P-GoSFET, as shown in Fig. 5b. The voltage amplifier showed excellent voltage amplification behavior, as presented in Fig. 5c. A maximum A_*V*_ as high as 9 dB was obtained at a load resistance (*R*_*D*_) of 50 kΩ (Fig. 5d).

In summary, the utilization of a silicon substrate in graphene FETs without an insulating layer leads to robust current saturation under wide-ranging and exceptionally high output resistance. In addition, the proposed GoSFETs show excellent process compatibility with conventional FET processes. This method potentially can enable the implementation of graphene in RF device applications.

## Methods

### Device fabrication

The graphene used in this work was synthesized using the inductive coupling plasma chemical vapor deposition method on a thin copper film, and it was transferred using the typical wet transfer method with PMMA. For the conventional GoIFETs, the graphene was transferred onto thermally grown SiO_2_ (100 nm) on a heavily doped p-type silicon wafer (resistivity of ~0.01–0.05 Ω∙cm). For the GoSFETs, the thermal oxide (50 nm) in the active region on lightly doped n- or p-type silicon (resistivity of ~1–5 Ω∙cm) was patterned using a buffered oxide etchant, and the graphene was transferred immediately after the removal of the photoresist and the native oxide. The subsequent processes were identical for both the GoSFETs and GoIFETs. After patterning the channel graphene, the source and drain were defined using a Pd (5 nm)/Au (50 nm) lift-off process. Next, 20 nm of Al_2_O_3_ was deposited by atomic layer deposition (ALD) process on the graphene surface. Prior to the ALD process, 0.5 nm aluminum layer was evaporated on the graphene surface as a nucleation layer. The ultra-thin aluminum nucleation layer is soon oxidized during the ALD process. The 150 nm Al gate metal was formed using a thermal evaporator and the lift-off process. Finally, the device went through a thermal budget of 300 °C for 12 hrs.

### Electrical measurement

All of the devices were measured at room temperature in ambient air with a B1500 parameter analyzer. In order to implement a common source voltage amplifier with a graphene–on–silicon transistor, the external resistances (*R*_*G*_ = 1 MΩ, *R*_*D*_ = 10, 30, 50 kΩ) and capacitor (*C*_*G*_ = 1 μF) set on a breadboard, as well as a DC supplier and function generator, were connected. The transistors were staged in a probe station and connected to the circuit with a biaxial cable. The input signal was sinusoidal with an amplitude of 20 mV and a frequency of 1 kHz.

## Additional Information

**How to cite this article**: Song, S. M. *et al.* Improved Drain Current Saturation and Voltage Gain in Graphene–on–Silicon Field Effect Transistors. *Sci. Rep.* **6**, 25392; doi: 10.1038/srep25392 (2016).

## Supplementary Material

Supplementary Information

## Figures and Tables

**Figure 1:**
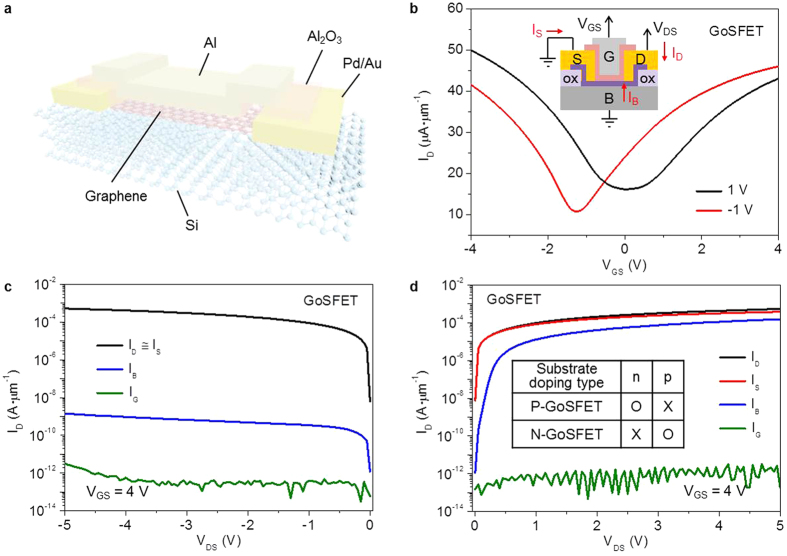
Graphene–on–silicon field effect transistors (GoSFETs) with W/L = 10/20 μm. (**a**) Schematic of a GoSFET with a lightly doped Si wafer acting as an insulator. The metal was patterned on top of the graphene. (**b**) Drain current (*I*_*D*_) versus top gate voltage (*V*_*GS*_) curves with positive (black) and negative (red) drain bias (*V*_*DS*_) on an n-type substrate. Drain current (*I*_*D*_), source current (*I*_*D*_), gate leakage (*I*_*G*_), and body current (*I*_*B*_) versus drain voltage (*V*_*DS*_) with *V*_*GS*_ = 4 V at the reverse bias condition of the Si substrate (negative *V*_*DS*_) in (**c**) and at the forward bias condition of the Si substrate (positive *V*_*DS*_) in (**d**). The table in **c** presents the proper GoSFET operating conditions depending on the substrate doping type.

**Figure 2:**
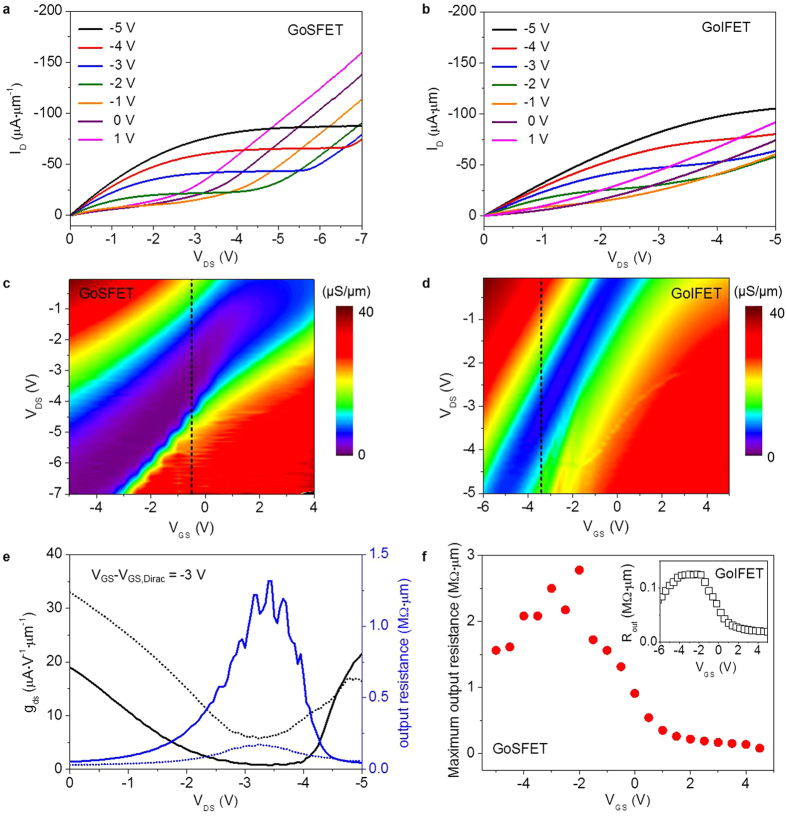
Output characteristics of GoSFETs and conventional GoIFETs. (**a**,**b**) The output characteristics of GoSFETs in (**a**) and GoIFETs in (**b**) at various *V*_*GS*_ − *V*_*GS,Dirac*_, where *V*_*GS,Daric*_ is the voltage at the minimum drain current near *V*_*DS*_ = 0 V. (**c**,**d**) The contour plots of the drain conductance (*g*_*ds*_) of the GoSFETs in **c** and GoIFETs in (**d**) show that the current saturation range of the GoSFETs is wider than the GoIFET saturation range. The dashed line indicates the condition of *V*_*GS*_ − *V*_*GS,Dirac*_ = −3 V. (**e**) Comparison of the *g*_*ds*_ of the GoSFETs (solid line) and GoIFETs (dotted line) at *V*_*GS*_ = −0.5 V and *V*_*GS,Dirac*_ = −2.5 V, directly showing the robust output characteristic of the GoSFET over those of the graphene FET. (**f**) The maximum output resistance (*r*_*o*_) of the GoSFETs and GoIFETs as a function of *V*_*GS*_, indicating that a higher *r*_*o*_ is obtained from the GoSFETs than from the GFETs.

**Figure 3:**
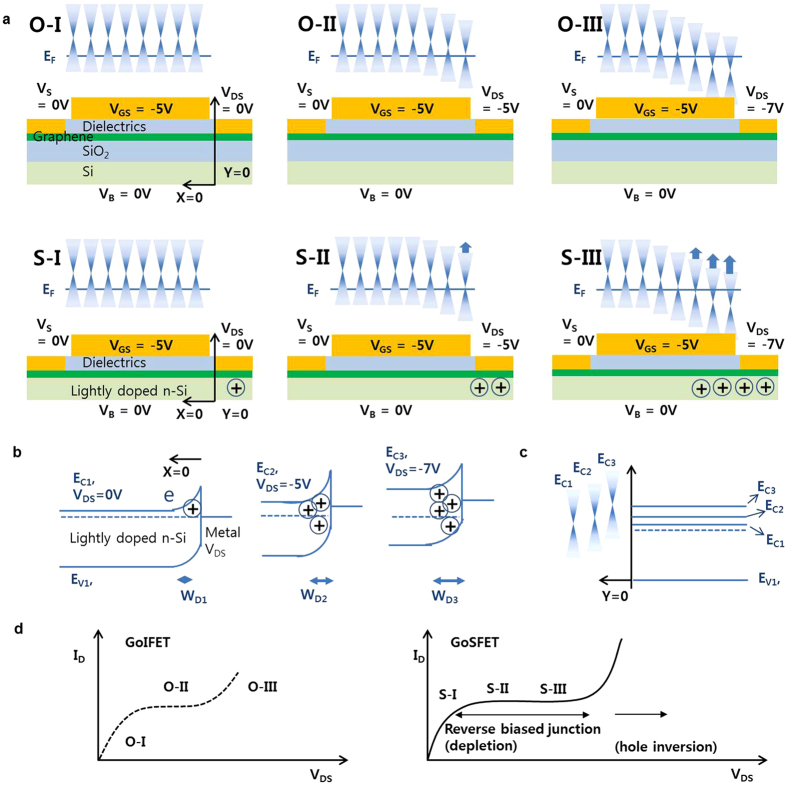
Conventional kink effect in a GoIFET and improved current saturation in a GoSFET. (**a**) A schematic demonstration of the energy band diagram of the graphene in both a GoIFET and GoSFET. The generation of an electron in the channel near the drain side under high drain voltage leads to a kink effect in the output characteristics of the GoIFET, while the fixed charge in the depletion region of the GoSFET allows the energy band diagram of the graphene to pull up so that the graphene is able to maintain its p-type behavior. (**b**,**c**) The pulling-up mechanism of the graphene energy band diagram, which is caused by the Si energy band in the GoSFET. (**d**) The measured output characteristics of the GoIFET and GoSFET, respectively. The sudden takeoff of the current in the GoSFET is steeper than that of the GoIFET because the current takeoff in the GoSFET is attributed to the body current (the depletion region disappears and the hole is inverted) rather than simply being due to the generation of the other carrier, as occurs with the GoIFETs.

**Figure 4:**
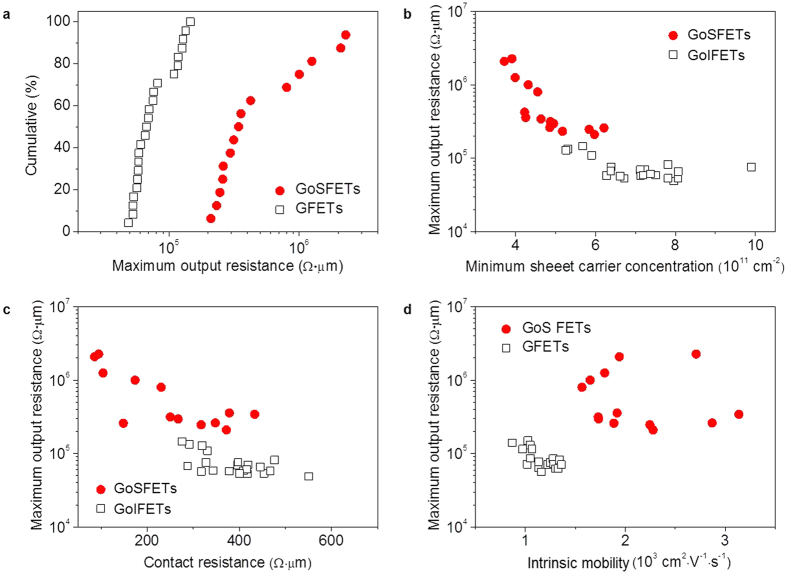
Comparison of GoSFET and GFET output characteristics. (**a**) Cumulative results of the maximum output resistance (*r*_*o*_), of the GFETs (black open squares) and GoSFETs (red filled circles). (**b**) Maximum *r*_*o*_ as a function of the minimum sheet carrier concentration (*n*_0_) of the GoSFETs and GoIFETs. (**c**) Maximum *r*_*o*_, as a function of the contact resistance (*R*_*C*_) of the GoSFETs and GoIFETs. The inset shows a schematic drawing of the potential profile of the graphene at the contact edge. (**d**) Maximum *r*_*o*_ as a function of the external mobility of the GoSFETs and GoIFETs.

**Figure 5:**
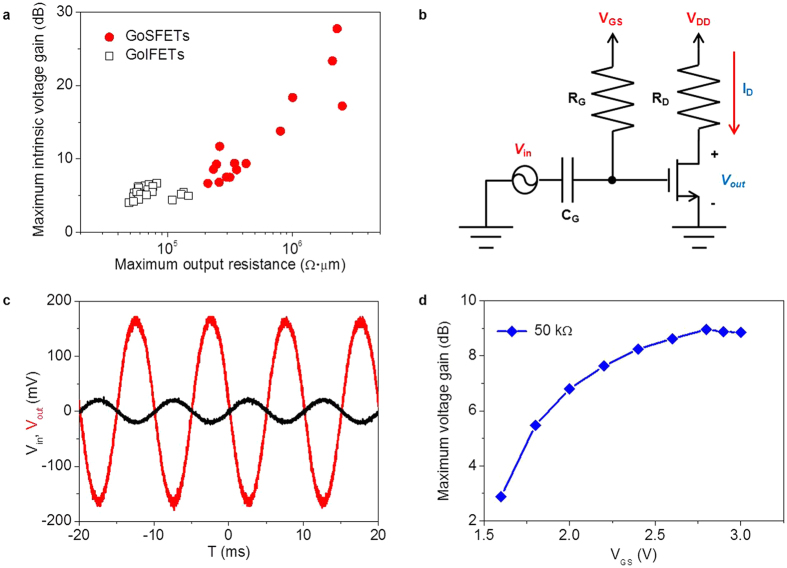
(**a**) Maximum intrinsic voltage gain (A_*V*0_) as a function of the maximum output resistance (*r*_*o*_) of the GoSFETs and GoIFETs. (**b**) A common source voltage amplifier schematic. One of the GoSFETs exhibiting a maximum intrinsic voltage gain of ~9.4 dB is applied to the circuit in order to measure the voltage gain (A_*V*_). The output voltage signal is obtained between the drain and ground. (**c**) Input (*V*_*in*_, black) and output (*V*_*out*_, red) signals measured using an oscilloscope. (**d**) A maximum A_*V*_ as a function of gate voltages at a load resistance (*R*_*D*_) of 50 kΩ.
