# Corrigendum: Improved Drain Current Saturation and Voltage Gain in Graphene–on–Silicon Field Effect Transistors

**DOI:** 10.1038/srep28412

**Published:** 2016-06-27

**Authors:** Seung Min Song, Jae Hoon Bong, Wan Sik Hwang, Byung Jin Cho

Scientific Reports 6: Article number: 25392; 10.1038/srep25392 published online: 05042016; updated: 07272016.

This Article contains errors in the Acknowledgements section.

“This work was supported by research grants from the National Research Foundation (NRF) of Korea (2010-0029132 and 2011-0031638).”

should read:

“This work was supported by the Center for Advanced Soft-Electronics funded by the Ministry of Science, ICT and Future Planning as Global Frontier Project (CASE-2011-0031638).”

