# Supplementary material for: Improved Drain Current Saturation and Voltage Gain in Graphene–on–Silicon Field Effect Transistors
Source: Sci Rep. 2016 May 4;6:25392. doi: 10.1038/srep25392 (PMC4855167; doi:10.1038/srep25392)
Supplement: Supplementary Information [file srep25392-s1.pdf]

Supplementary materials supporting the main manuscript

Improved Drain Current Saturation and Voltage Gain in Graphene–on–Silicon Field Effect Transistors

Seung Min Song[1], Jae Hoon Bong[1], Wan Sik Hwang[2], and Byung Jin Cho[1*]

[1]Department of Electrical Engineering, KAIST, Daejeon, 305-338 Korea,

[2]Department of Materials Engineering, Korea Aerospace University, Goyang, 412-791, Korea,

E-mail: bjcho@kaist.edu

1. Extraction of the residual carrier concentration, $\mathbf{n_0}$, contact resistance, $\boldsymbol{R_C}$, and FET carrier mobility, $\boldsymbol{\mu}$

The total device resistance, R_{Total}, is given by Equation (1). The values of n_0, R_C, and μ were extracted from the fitting the model.

$$R_{Total} = (2 \times R_C) + (\frac{\mathrm{L}}{\mathrm{W}})(\frac{1}{\sqrt{n_0^2 + (C_{OX} \times \frac{(V_{GS} - V_{GS,Dirac})}{q})^2} \times q \times \mu} \qquad (1)$$

where R_C is the contact resistance.

2. GoSFET's lower contact resistance compared to GoIFET

Figure S1 shows the X-ray diffraction patterns of the Pd/Si and Pd/graphene/Si stacks without and with the thermal budget. The reuslts show that the Pd easily reacted with the Si surface, forming Pd silicides even before the annealing process. Furthermore, the Pd was fully consumed to form Pd silicides after the annealing process, which was expected. However, surprisingly, the Pd silicide peaked in the Pd/graphene/Si stack before annealing, and the reaction was progressive after the annealing process.

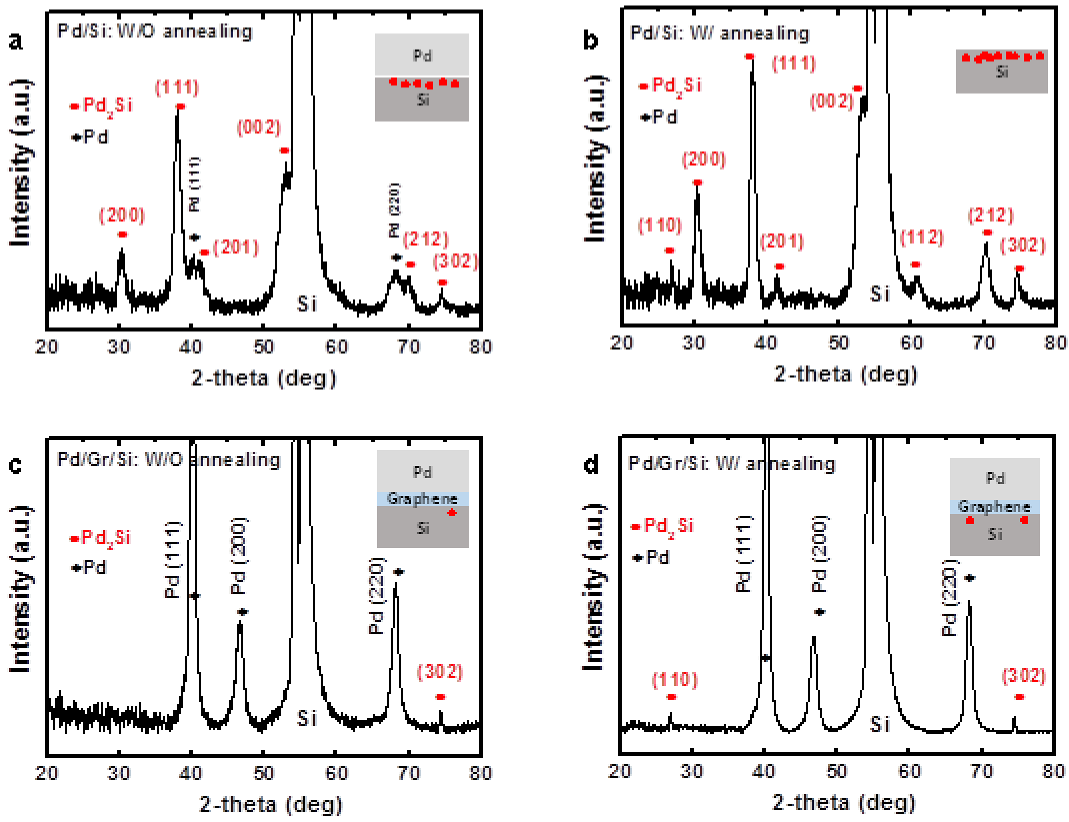

Fig. S1. Formation of Pd silicide on Si substrate. X-ray diffraction patterns of **a, b** Pd/Si stack without and with thermal budget, and **c**, **d** Pd/graphene/Si stack without and with thermal budget.

3. Raman anlysis of graphene on Si and SiO_2 surfaces

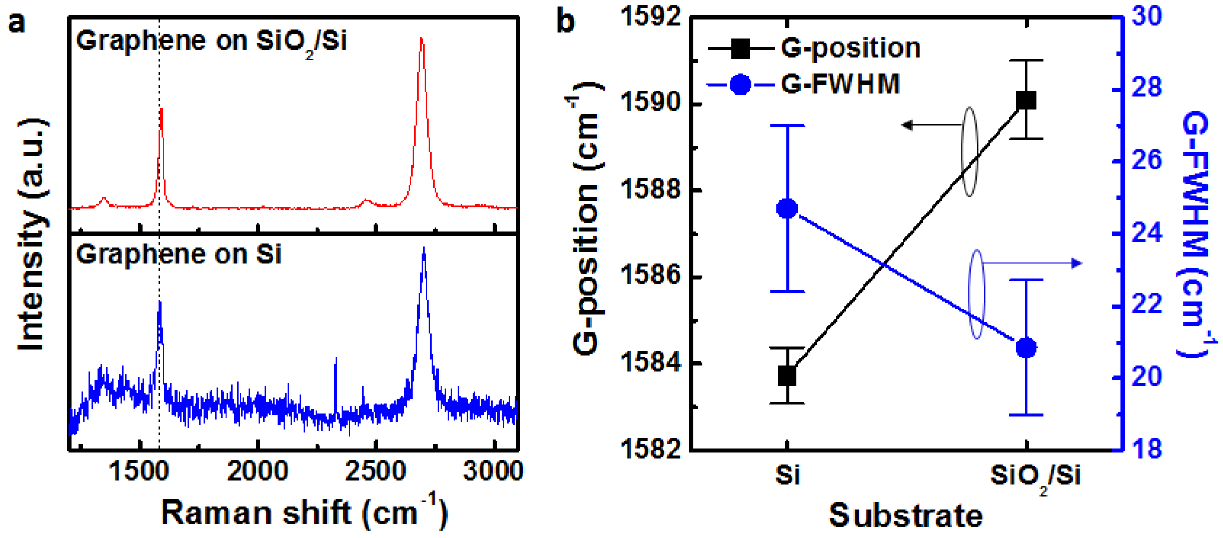

Fig. S2. a Raman analysis of the transferred graphene on SiO and Si substrates. **b** Position and full width at half maximum (FWHM) of graphene G peak depending on Si or SiO_2 surfaces. The excitation laser beam at a wavelength of 514 nm was focused onto the samples.

The residual charges in the graphene vary depending on different substrates, which can be assessed by Raman spectroscopy. The both position and full-width-at-half-maximum (FWHM) of G peak represent the carrier density in the graphene.[S1] Figure S2 shows Raman spectroscope of the transferred graphene on SiO_2/Si and Si substrates. The G peak of graphene occurs at higher position on SiO_2/Si substrate than Si substrate, and the FWHM of the G peak become narrower on SiO_2/Si substrate than Si substrate.[S1] The experimental results indicated that there are more residual charges on graphene/SiO_2 than graphene/Si interfaces.

References

S1. Pisana, S. et al. Breakdown of the adiabatic Born–Oppenheimer approximation in graphene. Nat. Mater. 6, 198-201 (2007).
